# The effect of internal and external focus of attention on tennis skill acquisition in children

**DOI:** 10.3389/fpsyg.2023.1308244

**Published:** 2023-11-30

**Authors:** Tuncay Tapan, Asuman Şahan, Kemal Alparslan Erman

**Affiliations:** ^1^Institute of Medical Science, Akdeniz University, Antalya, Turkey; ^2^Faculty of Sport Sciences, Akdeniz University, Antalya, Turkey

**Keywords:** tennis, focus attention, instruction, children, skill

## Abstract

**Objective:**

The aim of this study was to examine the effect of internal and external focus attention instructions on learning the tennis groundstroke (forehand-backhand drive) for children.

**Methods:**

A total of 60 (30 girls, 30 boys) children aged 10.24 ± 0.48 years were included in the study. Children were randomly divided into three groups: External Focused Group (EFG), Internal Focused Group (IFG), and Control Group (CG).

**Results:**

In the pre-training tests of tennis skill (TST) and tennis transfer (TTT), there was no significant difference between the three groups (EFG, IFG, and CG) according to one-way ANOVA results (*p* > 0.05). Significant interaction was determined between groups and measurements in a repeated-measures ANOVA analysis (three groups, three measurements) and TST and TTT (*p* < 0.01). According to the post-hoc analysis, it was determined that the TST results increased significantly in the EFG compared to the IFG and CG, and there was no significant difference in the TTT between the EFG and IFG, but both groups showed significant improvements compared to the CG.

**Conclusion:**

Instructions to children to focus attention externally facilitate learning the groundstroke (forehand-backhand) technique, which is one of the basic tennis techniques.

## 1 Introduction

Tennis is an open skill sport that involves complex movement and high levels of coordination. However, important cognitive and perceptual characteristics affect learning and performance in tennis (Tsetseli et al., 2016). Planning how to develop these essential characteristics in tennis during the learning process is of great importance for coaches and sports educators. Our research aimed to contribute to the literature by determining the effect of different approaches and reporting the effect of attention-focusing instructions on motor learning on effective and efficient learning in tennis.

Herein, the effect of internal and external attention focuses on tennis skill acquisition in children new to tennis was examined by comparing performance, learning, and transfer test results with a control group.

It is a common practice for coaches and teachers to give instructions during the learning of motor skills. These instructions can be associative (i.e., focusing on bodily sensation) or dissociative (i.e., blocking out sensations resulting from physical effort), broad or narrow, and external (i.e., on the effect of the movement) or internal (i.e., toward the body movement) (Niźnikowski et al., 2022).

There is evidence in the literature that instructions given toward the intended effect of the movements (external focus) rather than the movement itself (internal focus) provide more advantages for performance and learning (Wulf et al., 1999; Asadi et al., 2021). Many studies suggest that instructions with cues for the external focus of attention are better for athletes’ performance of motor skills than cues for internal attention focus (Wulf et al., 1998, 2000, 2007; Vance et al., 2004; Jackson and Holmes, 2011; Chiviacowsky et al., 2013).

It has been suggested that the athlete will use their decision-making abilities to choose the best motor response because the external focus of attention cues reduces the burden of working memory (Poolton et al., 2006). In a study that aimed to find the link between the breadth of attention and tactical decision-making, it was suggested that the reason team players could not find the most appropriate tactical solution in a game situation was that the coaches focused their attention in a certain direction by giving restrictive instructions (Memmert and Furley, 2007). It has also been suggested that the focus of attention during motor execution not only affects learning and performance but also increases movement productivity and efficiency (Wulf et al., 2010). In studies investigating the effects of using internal and external focus of attention on technical skill performance in vertical jump performance (Wulf and Dufek, 2009), jump performance (Chow et al., 2009), golf swing (Perkins-Ceccato et al., 2003), darts (Lohse et al., 2010), and basketball shooting performance (Al-Abood et al., 2002) external attention instruction conditions were found to have positive effects for external focusing.

The results of internal and external focus instructions for learning the dart throwing task were examined for 8- to 9-year-olds and 22- to 36-year-olds and a significant difference in performance between external focus and internal focus was found in the younger group. However, it was indicated that there was no difference between the groups that performed repetitions with internal and external focus instructions in children (Emanuel et al., 2008). However, in a recent study on table tennis players (12 females and 39 males with a mean age of 22.9 ± 1.8), it was stated that internal and external focus of attention had a similar effect on backhand accuracy on the development of low-skill players who had basic understanding and skills of table tennis strokes (Niźnikowski et al., 2022).

When physical education teachers teach forward somersault skills to primary school children aged 7 to 8, it has been observed that the skill develops more when they apply external-oriented instructions (Koufou et al., 2013). Abd Elahi states that boys between the ages of 8 and 14 were superior in terms of external focus of attention in performing a dribbling task under stressful conditions (Abdollahpour et al., 2008).

The participants of a study conducted by Porter et al. (2012) were 35 male university students with an average age of 22 and who had not engaged in athletics in their past or while at university. In the study, instructions were given to the external group to jump as close to a cone as possible; instructions for the internal group were to jump as far from the starting line as possible. The control group was instructed to make their best jump. The study suggested that the external group improved more in standing long jump performance compared to the internal and control groups.

Makaruk et al. (2013) examined the effect of external and internal focusing instructions on shot-put distance in their study on 30 national-level sprinters, jumpers, and shooters with an average age of 22. The results showed that the externally focused group achieved better results than the internally focused group. Milley and Ouellette (2021), in their study on 9 male and 16 female basketball players aged 18 to 24, investigated the effect of focus of attention during basketball free throw training. In this study, the use of verbal instruction produced better free throws in EFA imagery conditions than in IFA technique conditions.

In Silva et al. (2017) determined that the external group outperformed the internal group in the implementation, retention, and transfer stages in a study they conducted with 38 female athletes with an average age of 9.5 who were interested in ballet.

In another study, Hadler et al. (2014) conducted research with 21 girls and 24 boys with an average age of 11, asking them to hit the tennis ball forehand and backhand with their dominant arm. Participants had no previous tennis experience. The aim was to hit a target placed on the opposite side of the tennis court. The study examined the effect of external and internally oriented instructions and suggested that the forehand and backhand strokes of the external group showed better results than the other groups in the transfer tests.

Agar et al. (2016) included children aged 5–8 and 9–12 in their study on shuffleboard athletes. Their findings suggested that the group of older children performed better than the younger participants, but there was no significant difference between the externally focused and internally focused focus group performances during retention or transfer.

When the studies on the use of internal and external focus of attention and tennis are examined, it has been suggested that training using an external focus of attention increases the tennis game performance of children (Tsetseli et al., 2016). In a study examining the effect of internal and external focusing instructions on game performance in real match situations in 8–9-year-old tennis players, it was suggested that the externally focused group showed a significant improvement in decision-making compared to both the internally focused group and the control group. The study also said that the externally focused group showed better improvement than the other two groups in game performance and tennis-specific skills (Tsetseli et al., 2016).

Our study differed from that of Tsetseli and colleagues, in that internal and external focus instructions were given to children who were learning tennis for the first time, and specifically for instructions for each phase of the groundstroke technique. To determine the impact of attention, with a focus on motor learning, tennis targeting tests were used as the measurement method for all groups in the study to assess performance, learning, and transfer scores.

Our study aimed to examine the effect of internal and external focus of attention on groundstroke learning in tennis. We hypothesized that instructions given with an external focus of attention during tennis training would increase learning more than an internal focus of attention and training without instructions. To determine the effect of different focus of attention instructions on technical tennis learning, a control group (no instruction) was included in the study.

## 2 Materials and methods

### 2.1 Participants

A total of 60 children (30 boys and 30 girls) aged between 9 and 10 (10.24 ± 0.48 years) participated in the study. Participants had received tennis training for 2 months before the study. After obtaining permission from the University’s Ethics Committee, children and their families were informed about the purpose of the study and written consent was obtained. All children participating in the study were informed that taking part was voluntary and that they could leave the study whenever they wanted. The children were randomly divided into three groups, the IFG internal focus group (n = 10 boys, n = 10 girls), the EFG external focus group (*n* = 10 boys, *n* = 10 girls), and the CG control group (*n* = 10 boys, *n* = 10 girls).

### 2.2 Variables, instruments, and procedures

#### 2.2.1 Experiment design

All groups participated in tennis training for 60 min, 3 days a week for 8 weeks. All groups had the same 10-min warm-up and 10-min cool-down phases. Training that included forehand and backhand drive basic techniques was given to the EFG with instructions to focus their attention outward and to the IFG with instructions to focus their attention inward. The CG was not instructed to focus any attention during the tennis training.

In accordance with the rules of the International Tennis Federation for the age group of the children taking part (International Tennis Federation, 2012), training was given on an orange tennis court (18 × 6.5 m) with an 80 cm high net, and with low-pressure balls (red, orange, and green). During the training, we ensured the rackets used by the children were of an appropriate size for their age. The instructions given during the training were designed to teach the five phases of the groundstroke (handling, preparation, footwork, contact point with the ball, and finishing) (Christmas and Elliott, 2001). The training was in accordance with Gentile’s 2 × 2 matrix of skills classification (Gentile, 2000) and included ball-feeding and skills of increasing difficulty, given in four different contexts. Ball feeds were made as a transition from forehand and backhand hits made in a stationary position (closed), where the ball was dropped from the same height in each trial to forehand and backhand hits made in positions where the ball had various features (open). The same exercises were given to the children in all groups and with the same number of repetitions. All training was given by a Level 3 experienced tennis coach. A tennis skill test (TST) and tennis transfer test (TTT) were given to all groups pre- and post-training and after 2 weeks of having had no training (the retention test).

The internal and external attention-focusing instructions given for the teaching stages of the groundstroke technique are shown in Table 1.

**Table 1:** Teaching stages of the groundstroke.

Stroke phase	Instructions (External focus)	Instructions (Internal focus)
Grip	For forehand and backhand grip teaching, the child is asked to hold the racket by matching the line drawn on their hand to the line drawn on the handle of the racket.	Forehand grip: the child is asked to point their palm in the direction they will strike. Backhand grip: they are asked to point the palm down.
Shoulder rotation	The child is asked to turn their shoulder toward the net pole.	The child is asked to take their shoulder back.
Preparation	Before hitting the ball, the child is asked to draw the racket circle movement by opening the racket over the slalom pole placed in a T shape and bringing it under it. 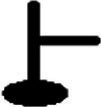 (slalom pole)	Before hitting the ball, the child is asked to draw the racket circle movement in the form of the letter C with their palm.
Footwork	The child is asked to step diagonally on the strip placed in front of them.	The child is asked to place their cross step forward.
Meeting the ball with the racket	The player is asked to hit the ball with the racket when it comes over the mini net placed in front of them.	They are asked to hit the ball at waist level in front of their step.
Finish	The child is asked to pull their racket up to the back after meeting with the ball and extend it.	After hitting the ball, they are asked to bring their elbow to eye-level.

Instructions were given at the beginning of each movement and remained the same for four training sessions for each phase of the movement. Immediately after the instructions, all players were required to perform 10 forehand or 10 backhand strokes.

#### 2.2.2 Instruments

**Figure 1:**
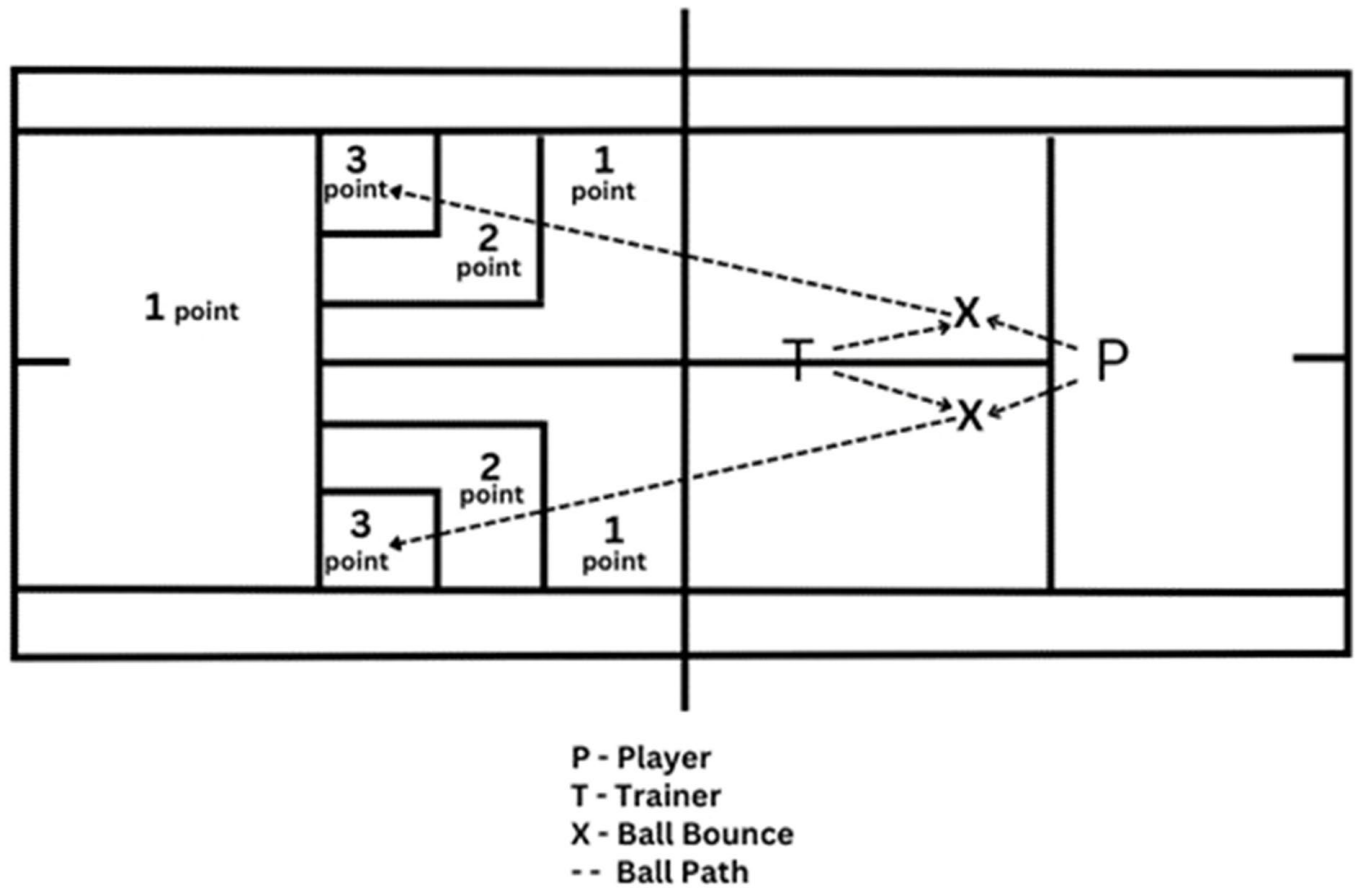
The tennis skill test.

**TST:** A total of 20 tennis balls were thrown by the coach to 10 forehands and 10 backhands of the player standing at the T point on the service line. The player hit these balls from 1 × 1 m and 2 × 2 m nested targets placed in counter service boxes to the highest scoring target by making 10 forehand and 10 backhand down-the-line hits. If the balls went out or were caught in the net, 0 points were awarded; if they fell inside, 3, 2, or 1 point were awarded according to the areas shown in Figure 1. The test was video recorded by the researcher and scored for each participant. If the balls landed on the border between two points, the higher score was recorded.

**Figure 2:**
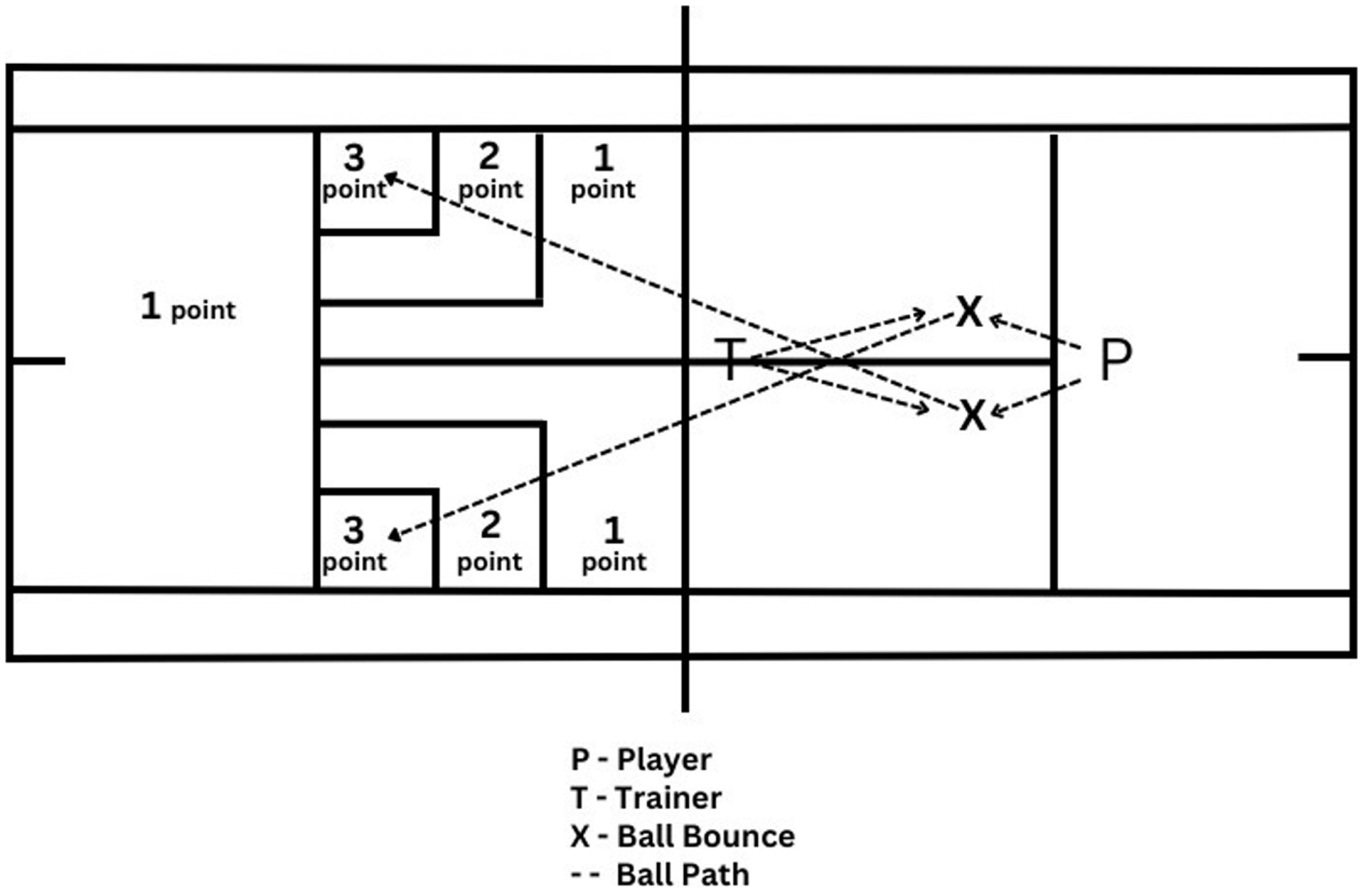
The tennis transfer test.

**TTT:** A total of 20 tennis balls were thrown by the coach to 10 forehands and 10 backhands of the player standing at the T point on the service line. The player hit these balls from 1 × 1 m and 2 × 2 m nested targets placed in counter service boxes to the highest scoring target by making 10 forehand and 10 backhand cross-court hits. If the balls went out or got caught in the net, 0 points were awarded. If they fell inside, then 3, 2, or 1 point were awarded as shown in Figure 2; each hit was scored live by a researcher. If the balls landed on the border between two points, the higher score was recorded.

The difficulty index of the 1×1 m and 2×2 m targets used in the TST and TTT was calculated with the formula developed by Paul Fitts (Fitts, 1954). Fitts’ law is an equation used to represent the time it takes for a target to reach an object. D is the distance from the starting point to the centre of the target, which is used to calculate the difficulty index, W is the width of the target, giving:

Difficulty Index = log2 (2D/W).

**Retention Test:** As a retention test, TST (including parallel shots) and TTT (including cross shots) were administered to all groups 2 weeks after the post-training tests.

### 2.3 Data analysis

Statistical analysis was carried out using IBM SPSS Statistics for Windows, version 22.0 (IBM Corp., Armonk, NY). Descriptive statistics and in-group distribution characteristics of the groups included in the study were examined. Time-dependent changes of the groups were determined by one-way in the comparisons of the pre, post, and retention tests between the groups, with the repeated-measures ANOVA test (three groups x three measurements) for more than two repeated measurements within the group. The significance level was taken as *p* < 0.05 and *p* < 0.01. The effect size was evaluated as *η*^2^ = 0.01 low, *η*^2^ = 0.06 medium, and *η*^2^ = 0.14 large effect level (Cohen, 1988).

## 3 Results

Overall, the children participating in the study were 10.24 ± 0.48 years, 132.35 ± 8.06 cm tall, weighed 34.42 ± 7.15 kg, and had an average BMI of 34.42 ± 7.15 kg/m^2^.

In the EFG:10.19 ± 0.47 years, height 127.45 ± 6.80 cm, weight 29.80 ± 4.60 kg, and BMI 29.79 ± 4.60 kg/m^2^.

In the IFG: 10.18 ± 0.48 years, height 135.70 ± 7.93 cm, weight 36.74 ± 7.50 kg, and BMI 36.74 ± 7.51 kg/m^2^.

In the CG: 10.33 ± 0.50, height 133.90 ± 7.29 cm, weight 36.72 ± 6.89 kg, and BMI 36.72 ± 6.89 kg/m^2^.

Table 2 shows there was no significant difference between the groups when comparing the scores obtained from the TST and TTT pre-tests of the children in the EFG, IFG, and CG (*p* > 0.05).

**Table 2:** Pre-test performance results of the tennis skill and transfer tests.

Variable	Group	M ± SD	*F*	*p*
TST (points)	EFG (*n* = 20) IFG (*n* = 20) CG (*n* = 20)	22.35 ± 4.64 22.35 ± 4.80 20.95 ± 4.45	0.61	0.55
TTT (points)	EFG (*n* = 20) IFGG (*n* = 20) CG (*n* = 20)	21.40 ± 5.65 22.00 ± 4.24 18.70 ± 4.12	2.77	0.07

According to one-way ANOVA *p* < 0.05.

Table 3 shows a significant difference in time-dependent changes in repeated measurements for the TST – *F* (1.52,86.73) = 56.81, *p* < 0.01.

**Table 3:** Performance scores of groups for the tennis skill test pre-, post-, and retention tests.

TST (point)	EFG	IFG	CG
Pre-test	22.35 ± 4.64	22.35 ± 4.80	20.95 ± 4.54
Post-test	28.10 ± 4.22	24.15 ± 4.72	21.80 ± 4.01
Retention test	27.05 ± 3.66	21.30 ± 3.97	19.20 ± 2.89
Measurement	*F*_(1,52;86,73)_ = 56.81	*p* = 0.0001**	*η*^2^ = 0.50
Group	*F*_(2;57)_ = 8.54	*p* = 0.0001**	*η*^2^ = 0.23
Measurement X group	*F*_(3,04;86,73)_ = 29.61	*p* = 0.0001**	*η*^2^ = 0.51

According to repeated-measures ANOVA analysis *p < 0.05; **p < 0.01.

When evaluated after ignoring the measurement variable, a significant difference between the groups was found – *F* (2,57) = 8.54, *p* < 0.01.

When the group and measurement interaction was examined, a significant difference was found between the groups – *F* (3.04,86.73) = 29.61 (*p* < 0.01).

As a result of the post-hoc analysis performed on the TST parameter, a significant difference was found between the EFG and IFG – *p* = 0.04 (*p* < 0.05). A significant difference was found between the EFG and CG – *p* = 0.0001 (*p* < 0.01). There was no significant difference between the IFG and CG – *p* = 0.39 (*p* > 0.05).

According to the results obtained from the ANOVA test based on the comparison of groups in the post-test for the TST, there was a significant difference between the EFG and IFG with *p* = 0.016 (*p* < 0.05). There was a highly significant difference between the EFG and CG with *p* = 0.0001 (*p* < 0.01). However, there was no significant difference between the IFG and CG with *p* = 0.273 (*p* > 0.05).

For the retention tests, a highly significant difference was found between the EFG and IFG with *p* = 0.0001 (*p* < 0.01). A highly significant difference was found between the EFG and CG *p* = 0.0001 (*p* < 0.01). However, there was no significant difference between the IFG and CG with *p* = 0.197 (*p* > 0.05).

Table 4 shows a significant difference in time-dependent changes in repeated measurements in TTT – *F* (1.70; 96.72) = 39.84, *p* < 0.01.

**Table 4:** Performance scores for the tennis transfer test pre-, post-, and retention tests.

TTT (point)	EFG	IFG	CG
Pre-test	21.40 ± 5.65	22.00 ± 4.24	18.70 ± 4.12
Post-test	26.35 ± 5.10	23.35 ± 3.38	19.95 ± 4.42
Retention test	24.75 ± 4.14	20.55 ± 2.09	17.85 ± 2.87
Measurement	*F*_(1,70;96,72)_ = 39.84	*p* = 0.0001**	*η*^2^ = 0.41
Group	*F*_(2;57)_ = 9.45	*p* = 0.0001**	*η*^2^ = 0.25
Measurement X group	*F*_(3,39;96,72)_ = 13.87	*p* = 0.0001**	*η*^2^ = 0.33

According to the repeated-measures ANOVA analysis *p < 0.05; **p < 0.01.

When evaluated after ignoring the measurement variable, a significant difference between the groups was found – *F* (2;57) = 9.45 *p* < 0.01.

When the group and time interaction was examined, a significant difference was found between the groups – *F* (3.39,96.72) = 13.87, *p* < 0.01.

As a result of post-hoc analysis performed in TTT, no significant difference was found between the EFG and IFG – *p* = 0.24 (*p* = 0.01). A significant difference was found between the EFG and CG – *p* = 0.0001 (*p* < 0.01). A significant difference was found between the IFG and the CG – *p* = 0.04 (*p* < 0.05).

According to the results obtained from the ANOVA test based on the comparison of groups in the post-tests for the TTT, there was no significant difference between the EFG and IFG with *p* = 0.101 (*p* > 0.05). There was no significant difference between the IFG and CG with *p* = 0.050 (*p* > 0.05). However, there was a highly significant difference between the EFG and CG with *p* = 0.0001 (*p* < 0.01).

Regarding the retention test, there was a highly significant difference between the EFG and IFG with *p* = 0.0001 (*p* < 0.01). A highly significant difference was found between the EFG and CG with *p* = 0.0001 (*p* < 0.01). There was a significant difference between the IFG and CG with *p* = 0.027 (*p* < 0.05).

## 4 Discussion

This study aimed to examine the effect of internal and external focus of attention instruction on the learning of the groundstroke technique in tennis. Herein, tennis targeting, retention, and transfer tests were applied at the beginning and end of the study to examine the learning of the tennis groundstroke technique of a control group and groups that trained with different instructions.

**Figure 3:**
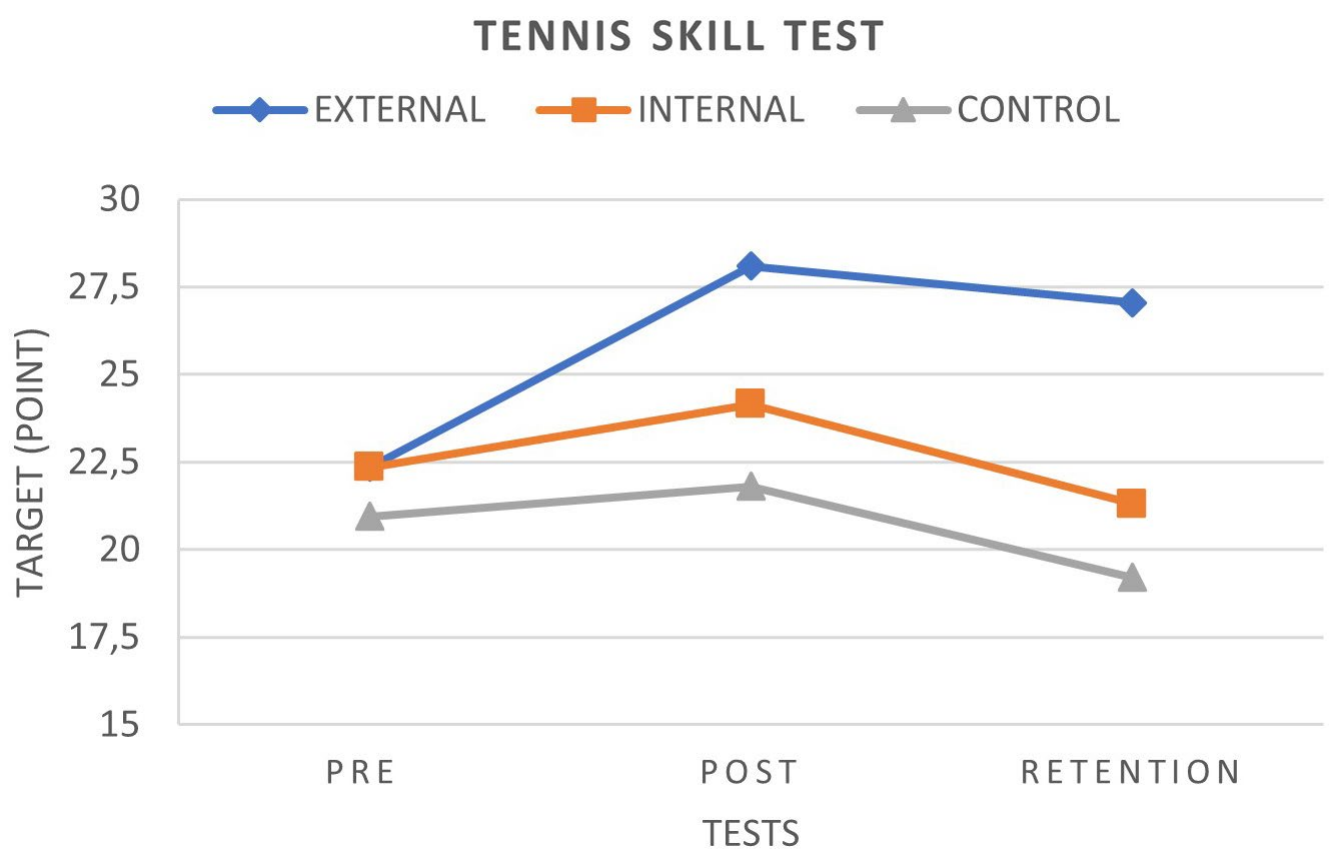
TST graph.

We found no statistically significant difference in the TST and TTT pre-test comparison results between the three groups (EFG, IFG, and CG) (*p* = 0.55 and *p* = 0.07). At the end of 8 weeks of training, the EFG showed significant improvement in TST compared to the IFG and CG. There was no significant difference in the development shown by the TST between the IFG and CG (Figure 3).

**Figure 4:**
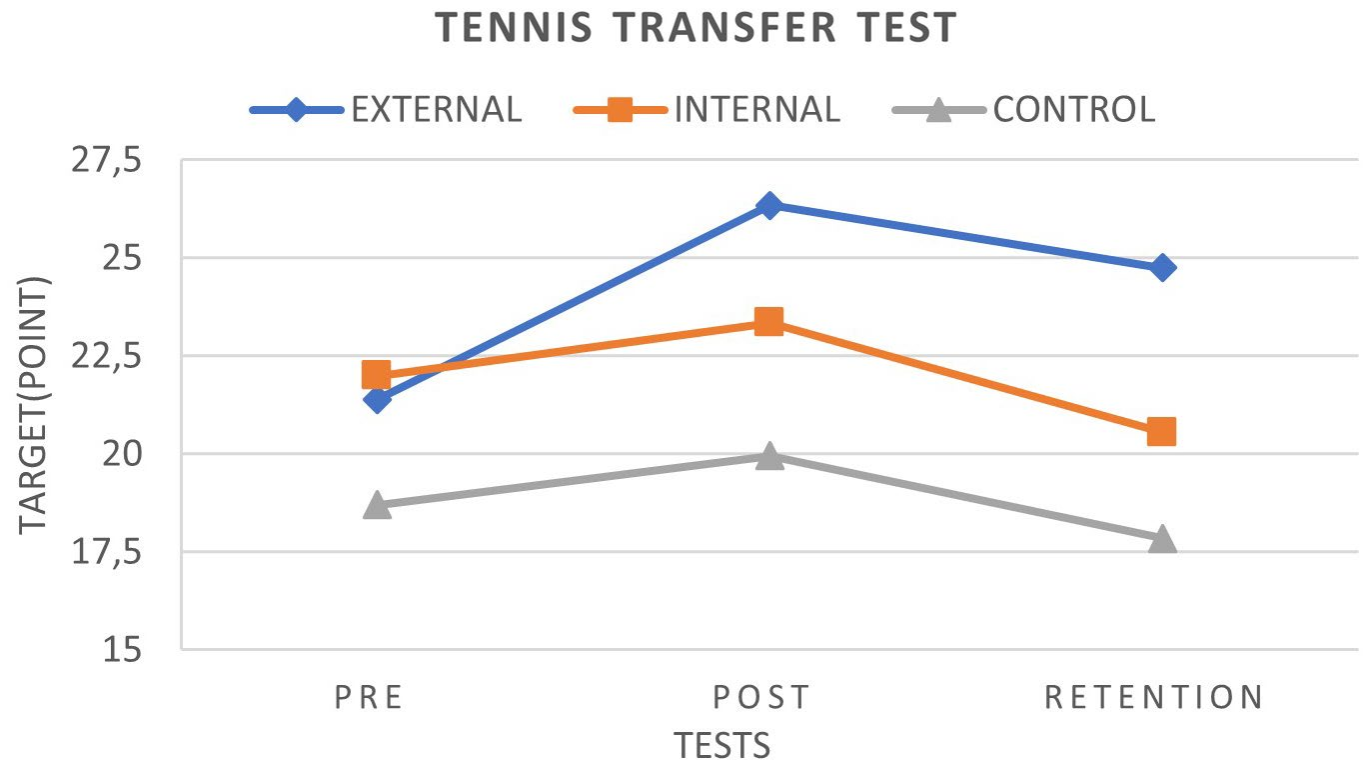
TTT graph.

We found no significant difference between the EFG and IFG for the TTT. However, there was a significant difference between the EFG and CG for the TTT. There was a significant difference between the IFG and CG in TTT (Figure 4).

At the end of the study, it was determined that the attention-directing instructions externally increased the learning and performance of the groundstroke technique more than the internal instructions and those who did not receive any instruction in child tennis players between the ages of 9 and 10. It was determined that there was no significant difference between the groups that received external and internal instruction in the transfer test, but both groups achieved significantly higher performance than the group that was not instructed (control group). According to these results, it can be said that focusing attention in tennis increases targeting performance, skill learning, and skill transfer. Furthermore, in this study, it was determined that the use of external focus instructions in tennis technical learning training in children improved performance and learning more than children who received internal focus instruction and did not receive instruction.

Our study does have some limitations. The results cannot be generalized to other sports, but this would be another avenue for future research. Future studies could explore the effect of externally and internally focused instruction on different tennis techniques. Future studies should include playing tennis in different age groups and ability levels.

## 5 Conclusion

The findings of this study indicate that external focus is more effective than internal focus in learning the tennis groundstroke for players who are new to tennis. These results suggest that tennis coaches and practitioners should make greater use of instructions that focus attention externally to facilitate motor performance.

## Data availability statement

The raw data supporting the conclusions of this article will be made available by the authors, without undue reservation.

## Ethics statement

The studies involving humans were approved by the present study received ethical approval from the T.C. Akdeniz University Sports Sciences Ethics Committee, as per their decision dated 19 June 2020, and designated with the reference number 377. The studies were conducted in accordance with the local legislation and institutional requirements. Written informed consent for participation in this study was provided by the participants’ legal guardians/next of kin.

## Author contributions

TT: Writing – original draft. AŞ: Writing – original draft. KE: Methodology, Writing – review & editing.
